# Topographical interrogation of the living cell surface reveals its role in rapid cell shape changes during phagocytosis and spreading

**DOI:** 10.1038/s41598-017-09761-6

**Published:** 2017-08-29

**Authors:** Maha A. Al Jumaa, Sharon Dewitt, Maurice B. Hallett

**Affiliations:** 1 Neutrophil Signalling Group, Sir Geraint Evans Building, Cardiff University School of Medicine, Heath Park, Cardiff, CF14 4XN UK; 2 Matrix Biology and Tissue Repair Research Unit, School of Dentistry, College of Biomedical and Life Sciences, Cardiff University, Heath Park, Cardiff, CF14 4XY UK

## Abstract

Dramatic and rapid changes in cell shape are perhaps best exemplified by phagocytes, such as neutrophils. These cells complete the processes of spreading onto surfaces, and phagocytosis within 100 s of stimulation. Although these cell shape changes are accompanied by an apparent large increase in cell surface area, the nature of the membrane “reservoir” for the additional area is unclear. One proposal is that the wrinkled cell surface topography (which forms micro-ridges on the neutrophil surface) provides the resource for neutrophils to expand their available surface area. However, it has been problematic to test this proposal in living cells because these surface structures are sub-light microscopic. In this paper, we report the development of a novel approach, a variant of FRAP (fluorescent recovery after photo-bleaching) modified to interrogate the diffusion path-lengths of membrane associated molecules. This approach provides clear evidence that the cell surface topography changes dramatically during neutrophil shape change (both locally and globally) and can be triggered by elevating cytosolic Ca^2+^.

## Introduction

**Figure 1:**
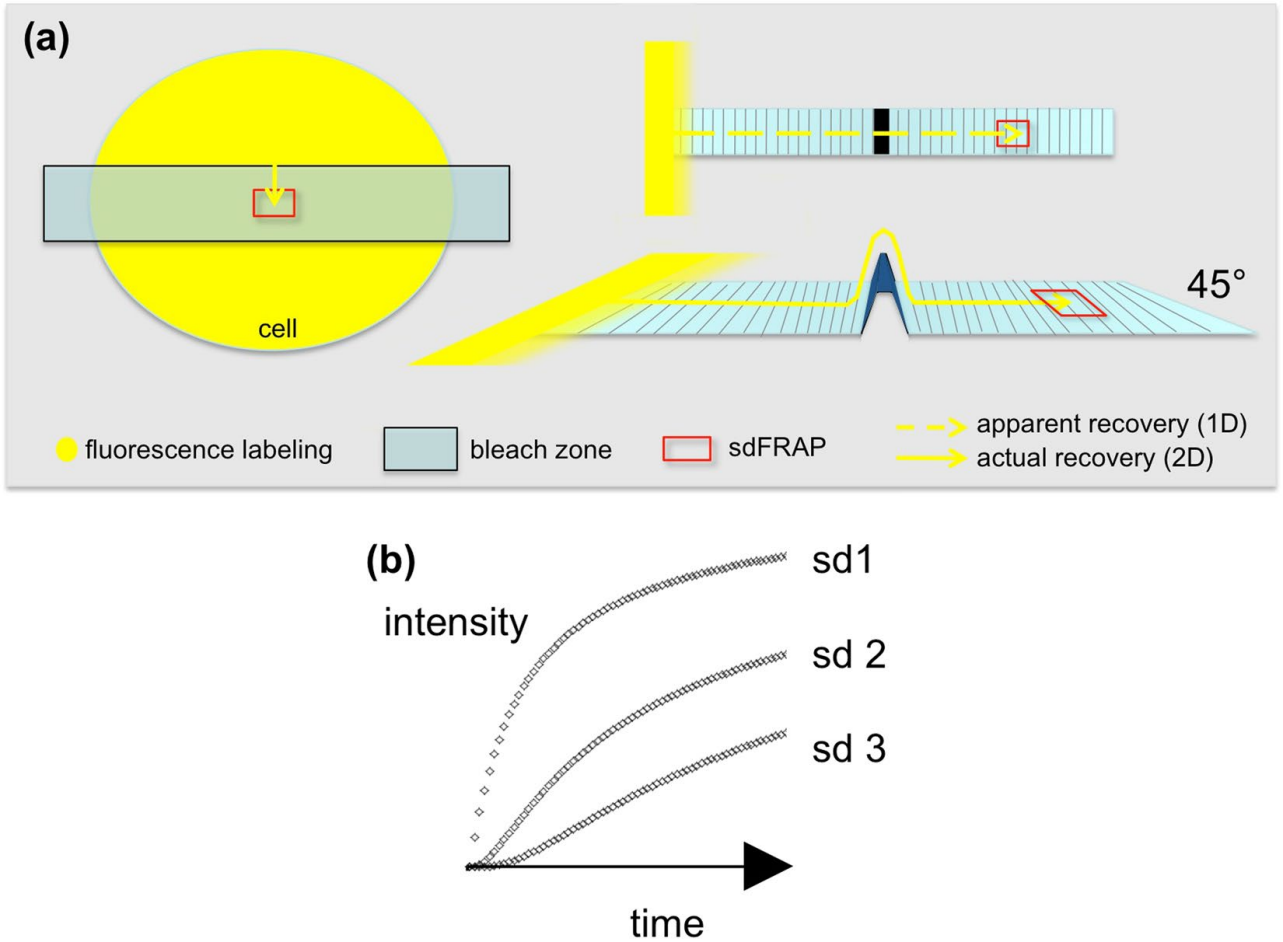
Principles of Subdomain FRAP. (**a**) The diagram illustrates the principle of sdFRAP applied to a fluorescently labelled cell membrane (left). The kinetics of diffusion into the subdomain (red box) from fluorescent molecules (yellow) from the edge of the bleached zone depends of the diffusion pathlength. The upper right part shows the apparent 1D pathlength (as viewed from above) in which surface topography is not apparent; the lower right shows the actual 2D (as viewed from a 45° angle), which includes topographical features. (**b**) The effect of distance on the recovery of fluorescence at a subdomain remote from the bleach front (sdFRAP) predicted from Fick’s law. The increase in fluorescence intensity with time at three subdomain distances (labelled sd 1, sd 2, sd 3) are shown. In the simulation, the intensity, time and distances shown are arbitrary but chosen to demonstrate the theoretical curves expected.

The lipid bilayers that make up the plasma membrane of living cells are strong transversely (ie across the water-lipid-water sandwich), but are intrinsically weak in the lateral direction and cannot stretch significantly without rupture^1^. Despite this, during phagocytosis by neutrophils or cell spreading, the surface area of the cell apparently doubles within 50–200 sec, suggesting that there must be a significant reservoir of membrane readily available for the increase in surface area^2, 3^. In some cells, intracellular vesicles may fuse with the plasma membrane when required^4^. It has been suggested that tension in the membrane regulates the addition of these vesicles ensuring that the tension is maintained but also prevented from exceeding the rupturing point^5^. However, there is evidence for an alternative source of additional membrane, namely that the wrinkled cell surface with its many micro-ridges may also form the basis for increasing the surface area, by the unwrinkling of these structures. Scanning electron micrographs of neutrophils undergoing phagocytosis or spreading, tantalisingly suggest that this explanation is tenable, as regions of the cell spreading out are devoid of such wrinkles^6^. A quantitative SEM study of macrophages undergoing phagocytosis correlated loss of surface wrinkles with phagocytosis^7^. However, such studies are based on a single time point post-event analysis and can be criticised because the fixation and preparation for SEM could induce an artefactual wrinkled appearance. More recently, a biophysical approach has shown that the wrinkled surface can be unwrinkled by applying suction through a micropipette, and that the force required to do so is reduced during phagocytosis^8, 9^. This points to a slackening of the forces holding the wrinkles in place during triggering of neutrophil shape change. However, all these studies give indirect evidence of the role for cell surface wrinkles, as it has not been possible to visualise or measure the changes in surface topography of living cells during these processes. In this paper, we report a novel experimental approach for gaining information about the wrinkledness of the cell surface of living cells. The new approach, which we have called subdomain FRAP (sdFRAP), monitors the rate of diffusion of a fluorescent marker molecule at a set apparent 1D distance into a zone photo-bleached of fluorescence. Differences in the timing of fluorescence recovery within the subdomain reflect the actual 2D diffusion distance that the molecules have travelled to arrive at the measurement subdomain. Obviously, topographical deviation from the planar would increase the actual 2D path length (Fig. 1). This difference in timing thus reflects the smoothness or wrinkledness of the path-length for diffusion. Using this approach, we show that non-spread living neutrophils have significant surface wrinkledness, which is lost (i) at the spread uropod tail during chemotaxis and (ii) locally near the phagocytic cup during phagocytosis, around the phagosome and extending pseudopodia. The surface topography can be altered experimentally by osmotically active media; by membrane expanders and by IP_3_-triggered Ca^2+^ influx, the physiologically relevant trigger^10–13^.

## Results

### Surface topography and subdomain FRAP

Although cell surface topography can be visualised by SEM (scanning electron microscopy), this approach does not allow for live cell measurement or dynamic changes to be investigated within the same cell. As SEM can only be used with cells that are fixed and gold-coated for imaging, it could be argued that such treatment would introduce wrinkles to cell surfaces or modify the cell surface so that it no longer reflected the true surface topography. In this paper we have developed a novel approach, which allows information of the cell surface topography to be extracted from living cells, and so permits dynamic changes in cell surface topography to be followed. This novel approach depends on measurement of the linear distance over which the diffusion of a membrane fluor had occurred (as if the surface were flat) and the actual diffusion path-length (i.e. including the surface contours). This approach was not intended to accurately measure diffusion constants. Instead, comparison of the recovery times of fluorescence at cellular locations (sub-domains) at the same geometrical configuration within a bleach zone before and after experimental treatment was used to obtain information about changes in diffusion path-length. Thus changes in recovery times rather than absolute values were used.

The diffusion of a lipophilic fluorescent marker incorporated into the plasma membrane from the edge of a region of plasma membrane denuded of fluorescence by photobleaching, to a defined site within the bleached zone, was monitored. The diffusion path length can thus be estimated by measuring the kinetics of fluorescence recovery at a location remote from the bleach front (Fig. 1). This is the principle underlying the use of subdomain FRAP (sdFRAP) to monitor cell surface topography. Unlike conventional FRAP, the rate of recovery of fluorescence is quantified at a defined 1D distance from the bleach front. As the time taken for diffusion depends on the distance that must be travelled, information can be obtained about the topography of the “terrain” over which diffusion has occurred. This effect can be seen by considering Fick’s law of diffusion, which can be simulated by dividing a linear section of membrane into equal compartments, with the same area of contact between compartments^14^. The flux in unit time from each compartment to the next is driven by the concentration difference across the boundary between compartments. The flux in unit time is given by Fick’s first equation J ∝(C2-C1) (where J is flux from adjacent compartments with concentrations C1 and C2). In this simple simulation, the effect of measuring the fluor concentration at distances remote from the bleach front can be seen (Fig. 1b), with delays in fluorescence recovery occurring at increasing distances, as well as differences in rates of recovery. Diffusion in a planar surface is described by D ∝x^2^/τ, where τ is the characteristic time and x is the distance travelled (x^2^ is the mean squared displacement at this time). It can be shown (see supplementary appendix) that the linear wrinkledness of the path length (actual 2D travel/apparent 1D travel) is given by **√**(τ_(w_)/(τ_(s)_) (where the τ is the characteristic time and subscripts, w and s, refer to wrinkled and smooth surface respectively). Thus the wrinkleness of the surface area is simply proportional to (τ_(w_)/(τ_(s)_), which we have used as a “topographical index” (Ti) such that a smooth surface would have a Ti = 1 and wrinkled surfaces have Ti values greater than 1. As the characteristic time (τ) is the reciprocal of the rate constant (k) for the recovery of fluorescence at the subdomain, the effect of changes in cell surface topography are reflected in changes in the rates at which fluorescence recovers at the subdomain site (see supplement B).

### Cell surface topography measured by subdomain FRAP

**Figure 2:**
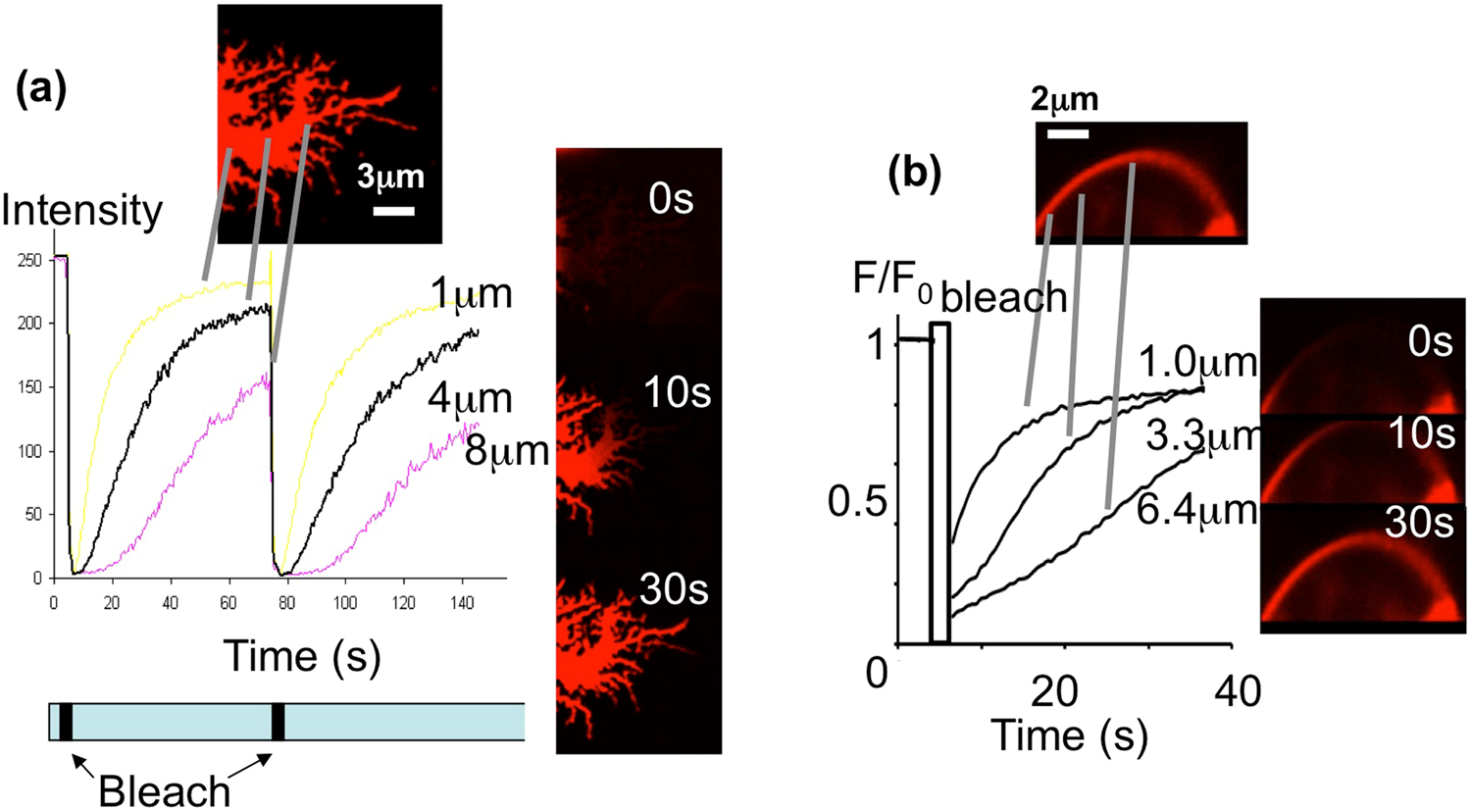
Demonstration of sdFRAP. Data from typical experimental situations are shown in which the diffusion distance of DiI molecules in the neutrophil membrane was sufficiently long that measurement from several subdomains could be taken. In both cases the upper images show the region of the cell before photobleaching; the lower graph shows the recovery of fluorescence intensity at the loci indicated (the distances from the bleach front are also shown); the series of images on the right show the recovery of fluorescence at the times indicated after photobleaching, for (**a**) the “flattened” neutrophil tail and (**b**) the osmotically swollen membrane. The data in (**a**) includes a second photobleach to show the reproducibility of these measurements. These data were typical of at least 3 other experiments in each case. The entire regions of the tail or swollen plasma membrane shown were bleached and subdomains within the bleached area of the tail were set at 1 μm, 4 μm and 8 μm front the bleach front; and in the swollen plasma membrane 1 μm, 3.3 μm and 6.4 μm. The complete data sets for (**a**) and (**b**) are shown as movies 1 and 2 in the accompanying supplementary data.

The tail region of highly spread and motile neutrophils is an extended, flattened region at the rear of the cell, which can extend over many microns (up to 15 μm). It provides a smooth membrane sheet that is ideal to test the theory of sdFRAP. A zone of the tail region of neutrophils previously loaded with the fluorescent membrane marker DiI (Dihexadecyl-3,3,3′,3′-tetramethylindocarbocyanine perchlorate, DiIC_16_(3)), was locally photo-bleached (Fig. 2a). The appearance of fluor at defined 1D distances from the bleach front was measured. The kinetics of recovery of fluorescence at defined distances were similar to those predicted by Fick’s simulation (Fig. 2a and movie 1). The recovery curves were used to estimate the actual (2D) distance travelled over an apparent 1D distance, and thus give a measure of wrinkleness of the tail membrane. It was found that the entire tail region of these cells had a homogeneous topography, with measurements of the diffusion constant (D) being approx. 1.43 μm^2^/s at all locations. This is significantly less than the measured diffusion constant reported for DiI (C_18_(3)) of 9.8 μm^2^/sec in artificial membranes^15^, but similar or higher than those reported in several other cell types, including erthrocyte ghosts which may have multiple spicules^16^ and has a reported D value for DiI (C18) of 0.2 μm^2^/s^17^. Since the D value estimated by sdFRAP in the neutrophil tail was constant regardless of the diffusion distance at which it was measured, it was concluded that there was no significant variation in surface topography in the tail region. In order to test whether this was the maximum rate for diffusion on a smooth (non-wrinkled) surface in our system, neutrophils were subjected to extreme osmotic swelling to generate fully bloated spherical cells (Fig. 2b; movie 2). In these cells, sdFRAP was again used to quantify diffusion at defined distances from the bleach front. The recovery curves were similar to those in the neutrophil tail region and gave estimates of D of 1.2 ± 0.3 μm^2^/s (n = 3), which is similar to the flat tail region. The slightly reduced D value suggested that the swollen cell membrane might not have been as fully extended as in the tail region.

In these experiments, the time delays in the arrival of fluorescent molecules within the recording subdomain were significant, as the diffusion length was over 10 μm. It would not be possible to achieve such large diffusion distances in other regions of the neutrophil (eg the phagocytic cup is only 2–3 μm diameter). Therefore, in the remainder of experiments reported here, the rate of recovery was measured at a defined location within the bleach zone (ie a sub-domain) for comparisons between different neutrophil states. After bleaching, the intensity increase can be fitted to an exponential curve (1-e^-kt^) with a rate constant, k, 0.17 s^−1^ estimated from the log transformation to give a linear relationship with a slope equal to –k (Fig. 1b). At a subdomain 2 μm into the bleached zone, k was found to be 0.13 ± 0.02 s^−1^ (mean ± sd). As the rate constant is the reciprocal of the “characteristic time” (τ), and D ∝x^2^/τ (where D is the diffusion constant and x is the distance), the characteristic recovery time was approx. 7.7 s. This can be compared to recovery of fluorescence within subdomains in the spread neutrophil tail (see previously), where the characteristic time at a 2 μm subdomain was approx. 2.86 s (giving a k value of 0.35 s^−1^). This analysis showed that in round (non-spread) neutrophils, the surface is significantly more wrinkled than in the tail of the spread neutrophil. In the appendix, it is shown that the diffusion path length compared to the flat tail will be given by *√*(τw/τs) and the wrinkled area is τw/τs (where τw and τs are the characteristic recovery times for the wrinkled and smooth surfaces). The sdFRAP data therefore shows that the non-spread neutrophil has a wrinkled topography that increases the diffusion distance on the cell surface by 1.6 fold and the surface area by 2.56 fold. Thus the cell body thus has wrinkles that would provide approximately 150% additional membrane which is sufficient to act as a reservoir for either cell spreading or phagocytosis^14, 15^. For simplicity, we have called the term τw/τs, the topographical index (Ti), as this indicates the apparent fold increase in diffusion area in 2D as a result of surface topography in 3D (see Fig. 1). It has been difficult to quantify this parameter in either fixed cells using SEM or AFM; or in live cells using AFM (see appendix E) and there are no previous reports. Our quantitation may thus represent the first direct measurement of the extent of the membrane “reservoir” afforded by cell surface topography in a living cell.

### Experimentally induced changes in cell surface topography

**Figure 3:**
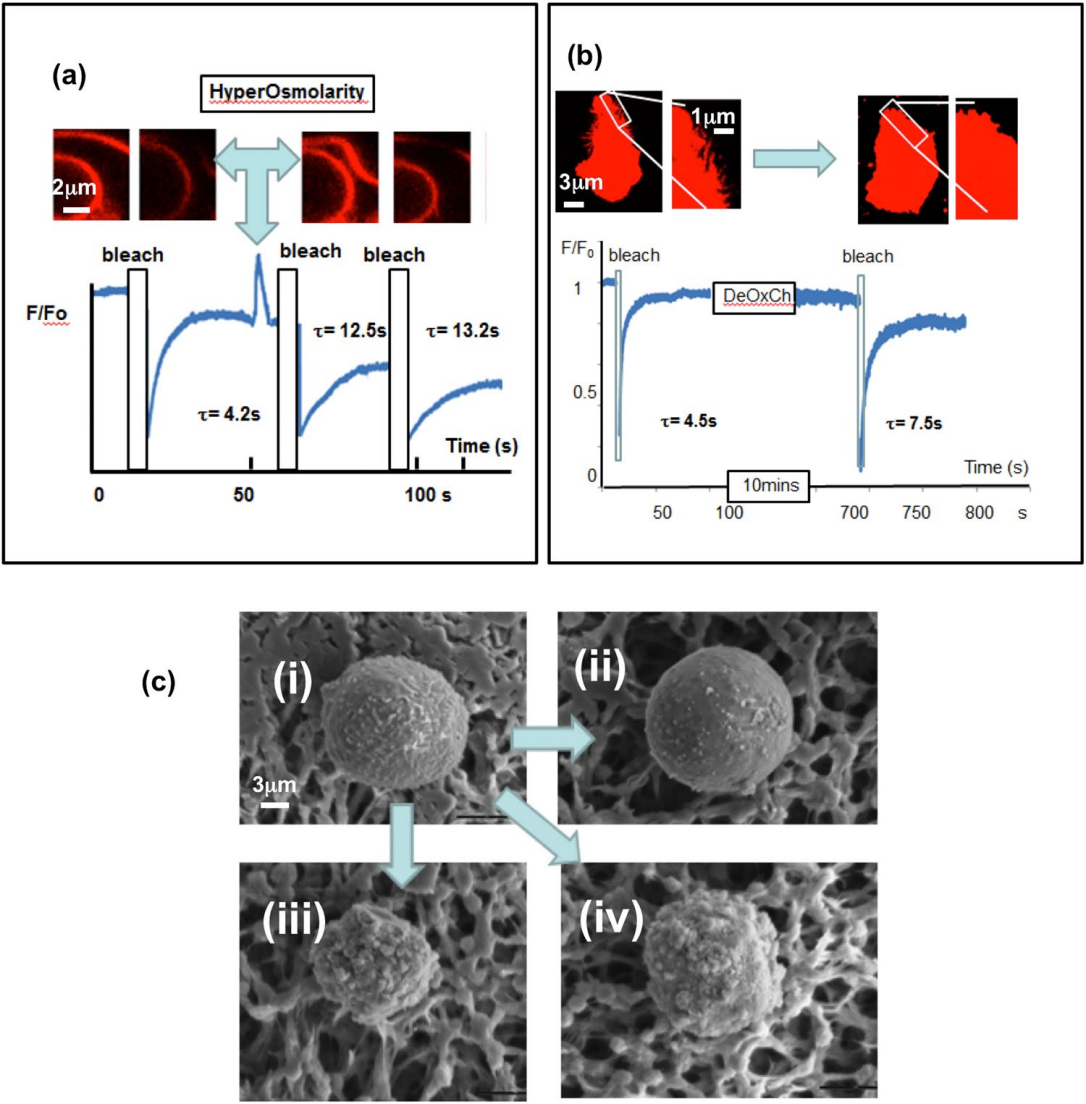
Experimentally induced changes in cell surface topography. (**a**) The effect of osmotic shrinking (600mOsM) on sdFRAP within a single neutrophil is shown. The pairs of images at the top show the DiI stained plasma membrane (and an internal nuclear membrane) initially and during photo-bleaching before and after osmotic shrinkage. The lower figure shows the complete time course of sdFRAP from three photo-bleaches of the same cell, one before osmotic shrinkage and two after shrinkage. The characteristic times for recovery, τ calculated from curve fitting, are shown for each sdFRAP. This experiment was typical of 3 cells, with 9 sdFRAP measurements. (**b**) The effect of deoxycholate on a single neutrophil is shown. The pairs of images at the top show the DiI stained cell at two magnifications before and after treatment with deoxycholate (10 mins at 10 mM). The lower figure shows the complete time course of sdFRAP from two photo-bleaches of the same cell, one before deoxycholate treatment and the other after. The time axis is broken during 10 min incubation as indicated by the label “DeOxCh”. The characteristic times for recovery,τ calculated from curve fitting are shown for each sdFRAP. This experiment was typical of 5 cells. (**c**) Scanning electronmicrographs of neutrophils treated as in the live cell experiments. (i) An untreated cell with numerous surface microridges (wrinkles); (ii) a cell after hypo- osmotic swelling with fewer surface wrinkles and increased cell volume; (iii) a cell after hyperosmotic treatment with a smaller cell volume and numerous additional cell surface features; (iv) a cell after treatment with deoxycholate showing additional cell surface features but a similar cell diameter. The micrographs were typical of similarly treated cells in the same microscopic field.

In order to investigate the factors that control the cell surface topography, the methodology was verified by experimentally induced changes. Initially, the effect of reducing the cell volume by osmotic shrinkage was investigated using either ionic (NaCl) or non-ionic (sucrose) hypertonic media. From the rate constants of sdFRAP recovery, the characteristic time was estimated to be approximately 33 sec (k = 0.03 s^−1^) giving a topographical index (Ti) of approximately 4.29, which is significantly higher than Ti = 2.6 in untreated cells. The effect could be demonstrated in the same individual cells before and after osmotic shrinking (Fig. 3a), where Ti similarly increased from 2.9 to 8.7 (k = 0.12 ± 0.2 s^−1^ to k = 0.04 ± 0.03 s^−1^ (mean ± sd, n = 3 cells) after osmotic shrinking. From the decrease in cell volume, it was estimated that the excess membrane formed the additional surface folds (Fig. 3c), which approximately agreed with the sdFRAP estimate (see appendix C). This provided a validation of the methodology and demonstrated the ability of neutrophils to accommodate osmotic shrinkage in hyperosmotic conditions (which may be experienced in the urinary tract and potentially at inflammatory cysts), by buckling their plasma membrane.

As a second validation, the surface area of the cell was experimentally expanded without significantly increasing the cell volume (in contrast to osmotic swelling) by the inclusion of lipophilic molecules, such as deoxycholate, into the plasma membrane^18^. Treatment of neutrophils to expand the plasma membrane in this way also produced a significant increase in diffusion path length (Fig. 3b), with the topographical index increasing to 4.2 ± 1.1 (n = 5). The experimental procedure added artificial membrane area, which was accommodated in additional cell surface topographical features such as micro-bloating and bloated wrinkles that were observed by SEM (Fig. 3c). The additional surface features provided contours in addition to the physiologically wrinkled topography.

Thus these simple experimental manipulations demonstrated the validity of the sdFRAP method for revealing cell surface topography of live cells at the sub (light) microscopic level.

### Changes in cell surface topography accompany phagocytosis

**Figure 4:**
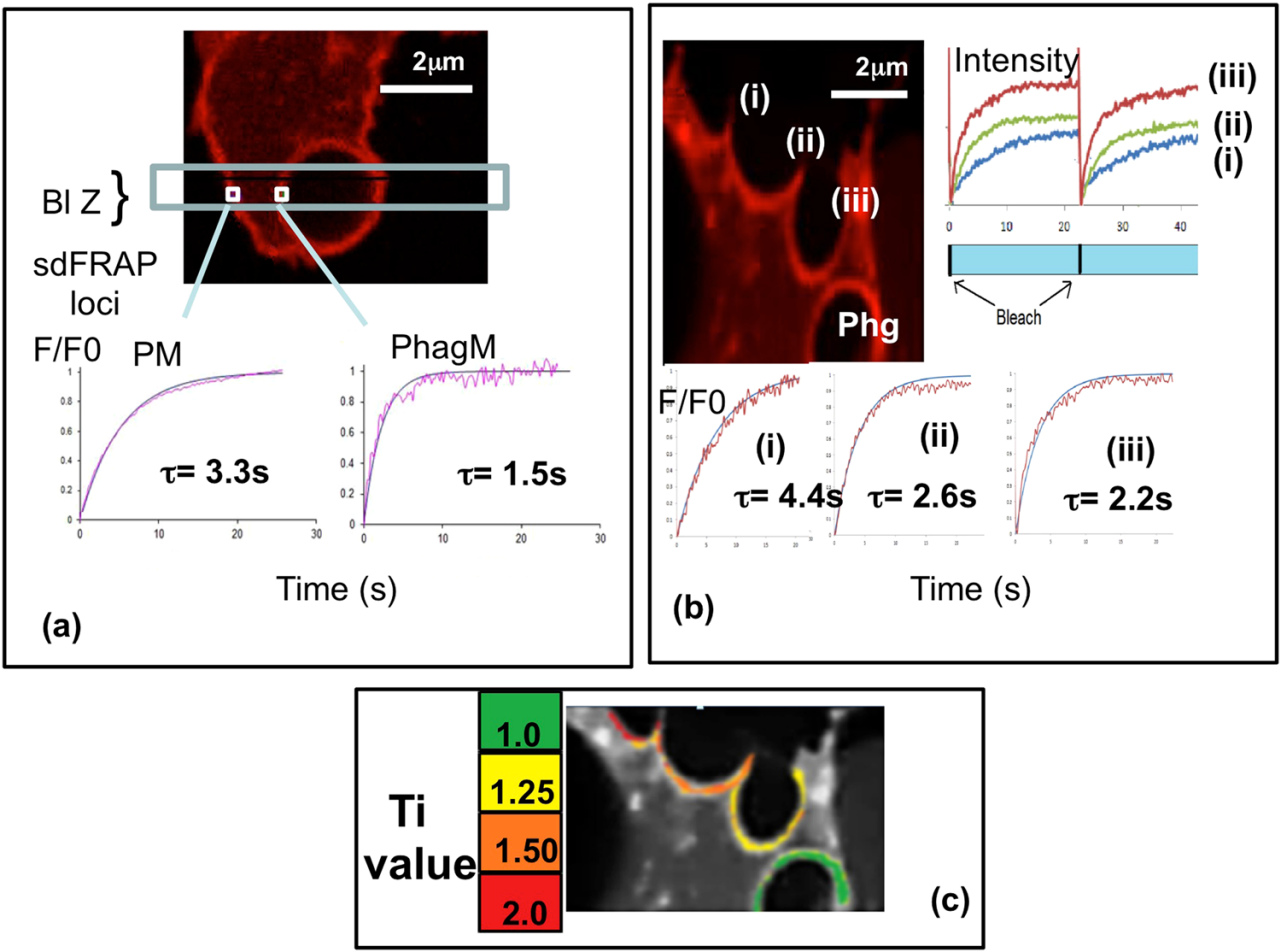
Cell surface topography during phagocytosis. (**a**) The image shows the DiI staining of a portion of a neutrophil which has undergone phagocytosis of an opsonised zymosan particle, with the photo-bleached zone (Bl Z) and the loci of two measurement regions (sdFRAP loci) marked. The graphs below show the sdFRAP recovery of fluorescence in the two regions ie the plasma membrane (PM) and the phagosomal membrane (PhgM) with the fitted curves from which the characteristic times, τ were estimated. This data is representative of at least 7 other phagosomes measured. (**b**) The image shows the DiI staining of a portion of a neutrophil which has completed phagocytosis of an opsonised zymosan particle (marked “Phg”), and has three phagocytic cups marked i, ii, and iii) en route to full phagocytosis. The graphs to the right shows the raw data from two photo-bleaches measured at three loci one each within the membrane of the three phagocytic cups. The graph below show the sdFRAP recovery of fluorescence in the three phagocytic cups with the fitted curves from which the characteristic times, τ, were estimated. (**c**) The phase contrast image of the cell shown in (**b**) has been overlayed with pseudo-colouring to illustrate the distribution of Ti values as a measure of the wrinkledness of the membrane, where a Ti value of 1 is smooth and higher values indicated increasing surface features. The colour bar shows the cut-offs used where green was used for Ti values of 1–1,25; yellow 1,25–1.5; orange 1.5–2.0 and red for greater than 2.0. This data was typical of three cells in which phagocytosis within an individual cell displayed three stages of phagocytosis.

The main function of neutrophils is phagocytosis. After completion of phagocytosis, phagosomes form which appear to conform to the shape of the particle. Subdomain FRAP measurements show that the phagosomal membrane is smooth, the actual diffusion distance being equal to the apparent 2D distance (topography index = 1.1 ± 0.2; n = 7); whereas the cell body of the same cell remained wrinkled with a topography index = 2. 5 ± 0.35; n = 7 sig diff p < 0.05 (Fig. 4a). In order to establish how this transition is achieved, we exploited the short pause in phagocytosis that occurs after phagocytic cup formation^8, 13^. This provided a short window sufficient to investigate the localised topography changes during the early events of phagocytosis (Fig. 4a). It was found that the membrane around the phagocytically active part of the cell had localised differences. The inner convex surface of the phagocytic cup was smooth (topography index = 1), whereas the protruding pseudopodia supporting the phagocytic cup had topography indices between 1.5–2.1 suggesting it was wrinkled but to a lesser extent than the cell body 3–4 μm distant (topography index ≈ 2.5). These measurements were consistent with localised flattening of the wrinkled surface providing the additional membrane for phagocytosis, but also suggest that unwrinkling of near-by membrane may also be required for completion. In neutrophils undertaking multiple phagocytic events, i.e. forming more than one phagocytic cup simultaneously, it was possible to measure the topographical change at various stages of phagocytosis within the same cell (Fig. 4b,c). Cells were selected which displayed three distinct stages of phagocytosis; (i) early before the phagocytic cup had fully formed,(ii) at the phagocytic cup stage and (iii) late, as the phagocytic cup was closing. In 3 out of 3 cells in which it was possible to see these three stages, the membrane in contact with the target phagocytic particle became progressively smoother with the topographical index approaching 1 as phagocytosis progressed. The inner surface of the phagocytic cup and the membrane nearby showed significant loss of topographical features (wrinkles) as the phagocytic cup was formed (Fig. 4c). For comparison, the index of wrinkledness has been colour-coded and superimposed on the image to provide a map of the topography of the cell membrane around the phagocytic site at stages of phagocytosis (Fig. 4c).

**Figure 5:**
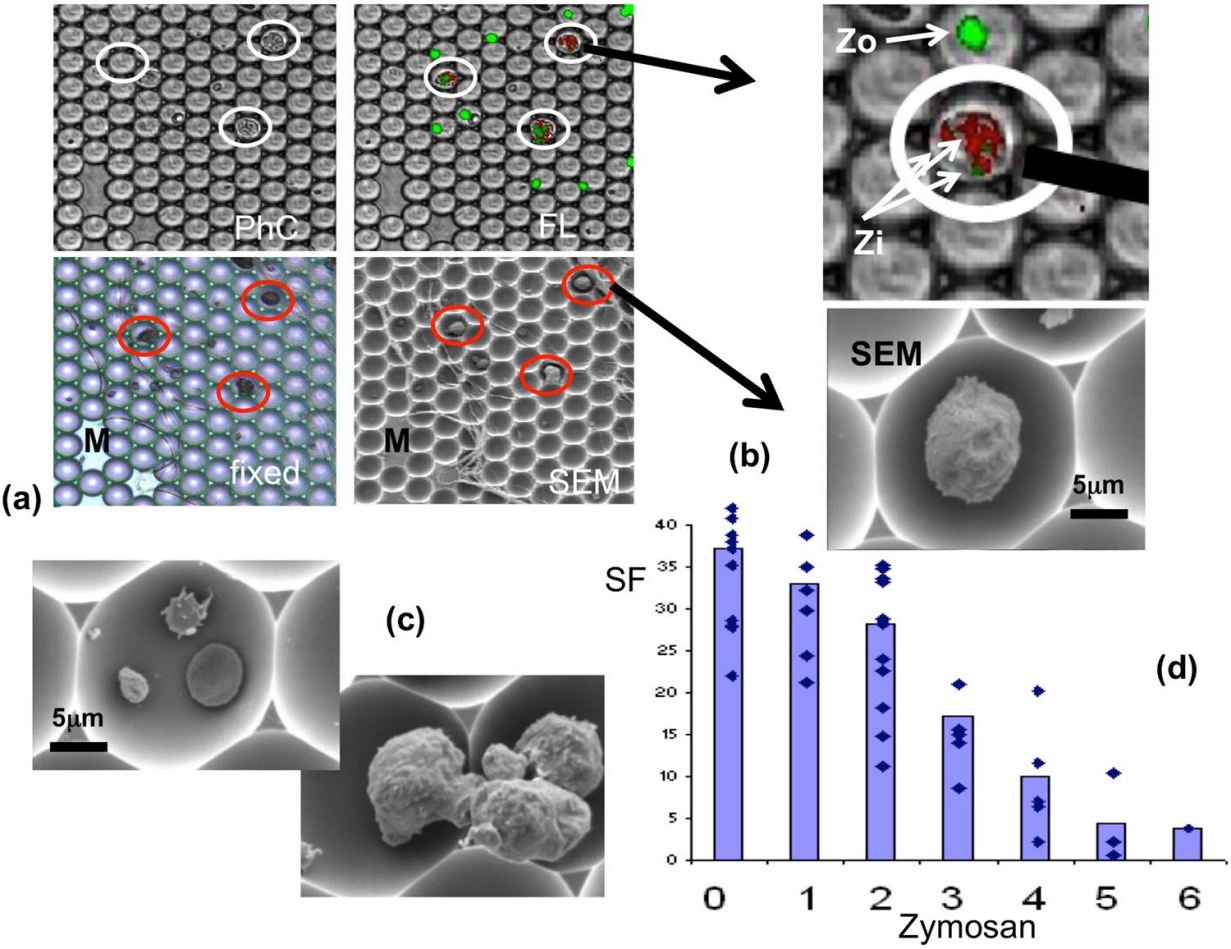
The relationship between cell surface features and phagocytosis. (**a**) The cell array containing live neutrophils (i) viewed under phase contrast (PhC) microscopy; (ii) the same region of the array viewed under fluorescent (FL) microscopy where the fluorescently labelled cells (red) and zymosan particles (green) can be seen; (iii) the same region of the array after fixation in preparation for scanning electron microscopy (fixed); (iv) the same region of the array viewed by scanning electron microscopy (SEM). There are markers which allow identification of individual neutrophils indicated, “M”. (**b**) A single identifiable neutrophil showed by an enlarged part of the cell array (upper image) and SEM (lower image). The fluorescent particles within the cells are dimmer (Zi) than the non-phagocyosed particles (Zo) and thus allow a distinction between cell associated and internalised fluorescent particles and can be quantified. (**c**) An example of an erythrocyte showing a smooth morphology in the same array pico-well as a platelet and a zymosan particle (upper image) and motile neutrophils with obvious wrinkled surfaces under the same conditions (lower image). (**d**) The relationship between the number of internalised zymosan particles, identified from fluorescence microscopy of the living cells, and the percentage coverage by surface features (SF) visible as bright highlights in the SEM images. Individual dots represent individual neutrophils (37 neutrophils were identified under both fluorescence and SEM imaging) and the bars show the means.

In order to test the conclusions drawn from of the novel sdFRAP measurements, a method was devised which permitted identification of individual neutrophils by light microscopy and fluorescence microscopy (to identify internalised fluorescently labelled particles) and the subsequent fixation and gold coating of the same cells for SEM imaging of the cell surface topography. This was achieved using LiveCell arrays (Nunc) to relocate individual neutrophils within identifiably located “picowells”. Cells which had undergone phagocytosis of fluorescently labelled zymosan particles were allowed to sediment into identifiable compartments of the array for light and fluorescence microscopy so that the number of internalised fluorescent particles by individual cells could be counted (Fig. 5a). The cells were then relocated (after fixation and gold coating) by SEM, so that the cell surface wrinkledness could be estimated (see appendix D) and quantified from surface contours evident on the SEM images (Fig b,c). It is unlikely that the fixation procedure and coating introduce wrinkles into membranes, as erythrocytes treated in this way were smooth and discoid as expected (Fig. 5c). This approach confirmed that there was widespread reduction of wrinkles on the cell body of neutrophils which had undergone multiple phagocytotic events and that there was a correlation between the number of internalised particles and the reduction of cell surface wrinkles (Fig. 5d).

These studies thus provide evidence both from live cells and fixed cells that the wrinkled cell surface topography of neutrophils provides the membrane reservoir for completion of the phagosome.

### Changes in cell surface topography during cell spreading

**Figure 6:**
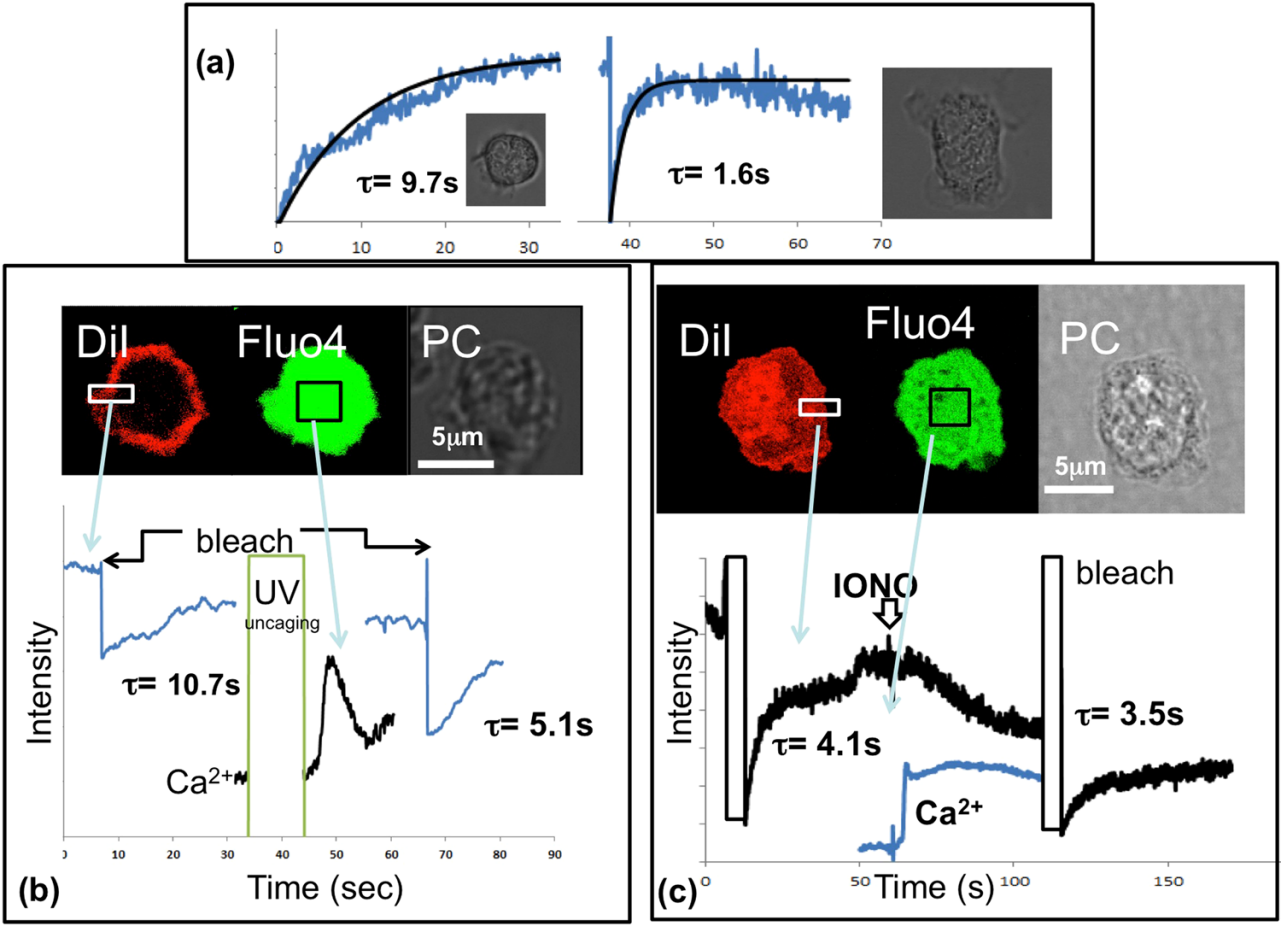
Cell surface topography during cell spreading and elevation of cytosolic Ca^2+^. (**a**) An example of the change in sdFRAP signal from an individual neutrophil before (left) and after (right) spreading on to a glass coverslip. The inset phase contrast images show the gross morphological difference in the cell before and after spreading. This was typical of at least 3 other similar experiments. (**b**) An example of a neutrophil loaded with the Ca^2+^ indicator fluo4, caged IP_3_ and DiI sedimented onto a plastic film coated coverslip to which it cannot spread. (i) The images show the DiI image (DiI) showing its location at the cell periphery, fluo4 showing its cytosolic location (fluo4) and the phase contrast image (PC). (ii) The graphs show the time courses of DiI fluorescence and fluo4 intensity. IP_3_ was uncaged by transient illumination with the 404 nm laser as shown (UV uncaging) while fluo4 intensity, as a marker of cytosolic Ca^2+^ was monitored. DiI was photo-bleached both before and after the IP_3_ –induced Ca^2+^ signal in the zone shown, and the characteristic times, τ for sdFRAP are shown. This was typical of at least 4 experiments. (**c**) An example of a neutrophil loaded with the Ca^2+^ indicator fluo4, and DiI, sedimented onto a plastic film coated coverslip to which it cannot spread. (i) The images show the DiI image (DiI) showing its location at the cell periphery, fluo4 showing its cytosolic location (fluo4) and the phase contrast image (PC). (ii) The graphs show the time courses of DiI fluorescence and fluo4 intensity. Ionomycin (4 μM) was added (IONO) while fluo4 intensity, as a marker of cytosolic Ca^2+^ was monitored. DiI was photo-bleached both before and after the ionomycin-induced Ca^2+^ signal, and characteristic times, τ for sdFRAP are shown. This data was typical of 3 experiments.

The second dramatic cell shape change which neutrophils undergo is spreading on a surface. This can occur on a glass surface (e.g. a glass coverslip) and occurs rapidly over a timescale of less than 100 s. It has been postulated that, as with phagocytosis, the additional membrane required for the transition from non-spread to spread is provided by the reservoir of membrane within the wrinkled surface^3, 11^. sdFRAP was therefore applied to cells in the process of spreading. It was found that in every cell, sdFRAP of the cell membrane of cells before and after spreading was significantly accelerated (Fig. 6a). The topography index of the cell body was reduced from 2.6 ± 0.4 to 1.43 ± 0.29 (n = 3) with a Ti value in unity with the tail region of all cells. This was consistent with the loss of surface wrinkles contributing to the additional membrane needed for spreading.

### Cell surface topography changes induced by transient Ca^2+^ influx

It has been known for 30 years that an elevation of cytosolic Ca^2+^ precedes the neutrophil spreading response^10, 19^ and that inducing a Ca^2+^ signal, triggers neutrophil spreading^11, 20^. Similarly, a Ca^2+^ signal is required for the localised cell shape change that is required for rapid phagocytosis^13, 21, 22^. The evidence points to a permissive role for Ca^2+^ signalling for neutrophil shape change, by the localised activation of calpain, a cytosolic Ca^2+^ activated protease which cleaves the cortical actin-plasma membrane linker proteins, such as ezrin^13^. While elevating cytosolic Ca^2+^ by photolytic uncaging of “caged Ca^2+^” can induce spreading^20^, this required a cytosolic Ca^2+^ level that is not physiological. In contrast, uncaging IP_3_ induces neutrophil spreading in response to physiological Ca^2+^ levels^13^. This is attributed to the indirect effect of IP_3_ by inducing Ca^2+^ influx^11^, which modelling suggests can elevate cytosolic Ca^2+^ within the wrinkles to sufficiently high levels to activate calpain^23^. As uncaging IP_3_ triggered a Ca^2+^ influx, which induced cell spreading, that itself alters the cell surface topography; in order to test whether an elevation of Ca^2+^ influx alone was sufficient to alter the cell surface topography, we used a plastic film substrate to which neutrophils cannot adhere or spread. The effect of photo-lytically uncaging IP_3_ in neutrophils under these conditions was established. It was found that an IP_3_ driven Ca^2+^ influx had a significant effect on sdFRAP in the absence of cell spreading (Ti = 4.0 ± 0.3 and 2.7 ± 0.4 before and after uncaging, n = 4: sig diff P < 0.05). After the Ca^2+^ signal, sdFRAP indicated a decreased diffusion pathlength (Fig. 6b), which would result from flattening of the cell surface wrinkles even in the absence of cell spreading. Interestingly, elevating cytosolic Ca^2+^ using ionomycin had no effect on the cell surface topography (Fig. 6c). This is consistent with the requirement for the opening of physiological Ca^2+^ channels, which generate high intra-wrinkle Ca^2^ as reported previously^11, 23^. Together these data point to Ca^2+^ influx being responsible for controlling the wrinkled morphology of the cell surface.

## Discussion

Although conventional FRAP is widely used to measure diffusion of lipid associated molecules, it is difficult to extract topography information from this. Aizenbud^24^ suggested on theoretical grounds that the topography of the surface could be ignored, as it would have little effect on the measured D value. In this model, the FRAP boundary increases with wrinkledness as well as diffusion path length. More recently, the effect of the surface terrain on the apparent D constant has been re-evaluated^25^ and modelling suggests that the relationship between the apparent D constant and the surface over which it is measured is relevant. Here we have taken a novel approach to deliberately make the diffusion pathlength the major factor, in order to extract information about the path length of diffusion.

The novel approach reported here depends simply on the incorporation of a fluorescent probe into the plasma membrane and monitoring recovery of fluorescence at a defined distance from the photo-bleach front (sdFRAP). This method clearly showed that the plasma membrane of neutrophils was significantly wrinkled, but that globally during cell spreading or locally in the neutrophil tail, this wrinkling is lost. Furthermore, during phagocytic cup formation and phagosome formation, the wrinkled plasma membrane becomes a smooth surface. These novel results are therefore consistent with the unwrinkling of the plasma membrane as being the reservoir of membrane in neutrophil shape change. This would explain the finding that ruffling of the neutrophil surface is inhibited if the cell is forced to adopt an extremely elongated morphology; and resumes when the cell is severed to form two non-elongated forms^26^. Our data suggests that the extremely elongated form takes up all the slack provided by the wrinkled topography, such that further deformation of the surface to form ruffles is prevented. On severing the cell, relaxation of the two cell fragments into non-elongated form would permit the slack in the membrane to become available again and the neutrophil would then resume surface ruffling. In that paper, the discussion was in terms of membrane tension. Membrane tension has also been implicated in both cell spreading^27^ and phagocytosis^28^. However, as ezrin is both a regulator of membrane tension^29^ and the maintenance of non-smooth cell topographies such as microvilli and microridges^30, 31^, membrane tension may be controlled by the ability to form these surface structures^29^ and so be intrinsically linked.

Additional important findings are that that the topography of neutrophils is modified locally during phagocytosis, and that the cell surface topography can be can be modified simply by uncaging cytosolic IP_3_, which releases stored Ca^2+^ and opens Ca^2+^ influx channels at the plasma membrane. This experimental procedure reduces the wrinkledness of the cell surface significantly even in cells which are prevented from spreading onto a surface. This finding explains why Ca^2+^ influx permits an increase in the rate of both neutrophil spreading and phagocytosis, as it makes wrinkled surface membrane available for the increase in surface area required. We therefore propose that cell surface topography, while previously little considered, is a controlled feature of cells which is a key element in maintaining cell surface tension and permitting the apparent membrane expansion required for spreading and phagocytosis.

## Methods

### Neutrophil isolation

Neutrophils were isolated from human blood taken from healthy volunteers with informed consent under Ethics Approval SMREC 10/01 (Cardiff University), and suspended in Krebs medium (120 mM NaCl, 4.9 mM KCl 1.2 mM KH_2_PO_4_, 1.2 mM MgSO_4_, 1.3 mM CaCl_2_, 25 mM HEPES and 0.1% BSA, adjusted to pH 7.4 with NaOH as previously described^32^. Neutrophils were allowed to sediment onto a glass coverslip for observation by confocal microscopy at 37 °C. Care was taken to avoid inadvertent activation of the cells, which can occur during shaking or vigorous re-suspension. All experiments were performed in accordance with relevant guidelines and regulations.

### Loading plasma membrane with DiI and FRAP measrement

Neutrophils were stained with 1,1′-Dihexadecyl-3,3,3′,3′-tetramethylindocarbocyanine perchlorate, DiIC_16_(3), dissolved in DMSO (10 mg/ml) either by dilution (to 2 μM) from the stock solution in cell suspension before addition of neutrophils to imaging chambers; or by addition of physiological medium (Krebs medium) to which DiIC_16_(3) (2 μM) had been added immediately before use, to coverslips onto which neutrophils had attached. After 120 s, the excess DiIC_16_(3) was removed from adherent cells by washing, i.e. replacing the dye-containing extracellular medium with media at least 4 times. Imaging was achieved using a resonant scanning laser confocal microscope (Leica SP5) with low intensity excitation at 543 nm to excite DiIC_16_(3) and an argon laser line at 488 nm used for photo-bleaching. The emitted light was collected for imaging at 600–700 nm. All experiments were performed at 37 °C. The PMT voltage on the detectors were set to give a high signal to background signal, resulting in a zero signal after bleaching. However, the background fluorescence signal from non-cellular regions of the image was subtracted before estimation of recovery times if necessary. The bleaching pulse (1 s) was restricted to a defined region of the cell producing a bleached zone at the membrane. This resulted in a decrease in total cell fluorescence by approximately 10%. As it is important to take this decrease into account when estimating recovery^33^, the recovery of fluorescence was allowed to continue until a new equilibrium signal was achieved. The rate of recovery to this equilibrium value was used to estimate diffusion distance at a subdomain within the originally bleached zone. Subdomains were measuring regions within the bleach zone 100 nm × 100 nm, usually taken 1 μm from the bleach front.

### Induction of phagocytosis

Zymosan particles (cell walls of saccharomyces cerevisiae) were opsonised by addition to freshly prepared human serum and incubated at 37 °C for 30 mins. The zymosan was then washed repeatedly by centrifugation to remove the activated serum (containing C5a). The zymosan suspension was added to neutrophils while observing microscopically. Contact between the neutrophils and the particles was allowed to occur by random chance and then followed as phagocytosis proceeded. Alternately, zymosan particles were allowed to adhere to glass coverslips by placing a droplet of zymosan suspension on a clean coverslip and left at room temperature for 15 mins. Adherence was monitored at the time and extended if necessary. Adherent zymosan particles were easily identified by the lack of Brownian motion indicating firm adherence. Non-adherent zymosan particles were washed away by the addition of excess medium with the aim of producing a sparse coverage of adherent zymosan particles (about 1–2 zymosan particle/400 μm^2^). The neutrophil suspension was then added to the coverslip while observing microscopically. After neutrophil adherence, human serum (1/10 dilution) was added. This results in both opsonisation of the zymosan (with iC3b) and the generation of C5a, which attracts neutrophils to the target.

### Scanning electron microscopy

Neutrophils, either in suspension or after sedimentation into cell array pico-well arrays were fixed with glutaraldehyde and gold coated as described previously^34^. From these images, the percentage of the cell surface occupied by surface features was estimated as previously described^35^ (and in supplementary appendix D). Essentially, pixels on the SEM images of cells which exceeded a threshold, set to distinguish high points on the cell surface which gave high intensity pixels from the remainder of the cell, were counted using Image J (NIH). The coverage of high intensity features on individual neutrophils was calculated within the same field using the same intensity threshold.

### Materials

DiI (1,1′-Dihexadecyl-3,3,3′,3′-tetramethylindocarbocyanine perchlorate) was purchased from ThermoFisher Scientific; caged-Ins(1,4,5)P_3_ /PM (D-23-O-Isopropylidene-6-O-(2-nitro-4.5-dimethoxy)benzyl-myo-Inositol 145-trisphosphate-Hexakis(propionoxymethyl) ester from Alexis Biochemical, U.S.A.; fluo4-acetoxymethyl ester from Molecular Probes Invitrogen, U.S.A; and all other reagents from Aldrich-Sigma. LiveCell arrays were purchased from Nunc Thermo Fisher Scientific.

### Data availability

The data generated during and/or analysed during the current study are available from the corresponding author on reasonable request.

## Electronic supplementary material

Supplementary Appendix

Movie 1

Movie 2
