# Supplementary material for: Topographical interrogation of the living cell surface reveals its role in rapid cell shape changes during phagocytosis and spreading
Source: Sci Rep. 2017 Aug 29;7:9790. doi: 10.1038/s41598-017-09761-6 (PMC5575107; doi:10.1038/s41598-017-09761-6)
Supplement: Supplementary file 1 — Supplementary Appendix [file 41598_2017_9761_MOESM1_ESM.docx]

**Topographical interrogation of the living cell surface reveals its role in rapid cell shape changes during phagocytosis and spreading.**Maha A Al Jumaa, Sharon Dewitt, and Maurice B Hallett

**Supplementary Appendix**

**A. Ficks diffusion simulator**

A section of membrane, divided into equal compartments, with the same area of contact to the next such that the flux in unit time (J) from each compartment to the next is driven by the concentration difference across the boundary between compartments, follows Fick’s Law: J∝(C2-C1) where J is the flux from one compartment to the next and C1 and C2 are the concentrations in each compartment.

The concentrations at each unit step in time in each membrane compartment (distant from the diffusion front) was thus calculated and the change in concentration over time plotted for three subdomains (see Fig 1b).

B. **Relationship between diffusion rate to subdomain and cell surface topography**

The diffusion from a constant source (of n_0_ molecules) into the bleached zone follows the following relationship ^1,2,3^:

where the concentration of molecules (n/n0) at point x from the source at time t from onset of the concentration boundary.

Using the first 2 terms of the Taylor series to approximate the error function, gives the following simple expression.

The concentration of fluor (or intensity as F/F0) after photobleaching within the subdomain at a distance x from the bleach front is thus given by

Thus an intensity (conc of fluor molecules) reaches a fraction of F_0_ (ie F/F_0_=f) within a subdomain at distance of x when time is t

**x^2^= (1-f)^2^. π. Dt**

Thus the time for the intensity to rise to a defined fraction of the final concentration is in proportion to the square of its distance from the source. This is a fundamental property of diffusion. The

the time to reach ½ (half time, t_1/2_) or ( 1/e)^th^ (characteristic time τ) of the equilibrium concentration is given by x^2^= (π/4) Dt_1/2_; and x^2^= (0.4)π .Dτ. Both obey the relationship x^2^ ∝ t .

The relationship x^2^ ∝ t for sdFRAP recovery at subdomains at defined distances times can be shown experimentally. In this example below, the plasma membrane of the tail region of a spread neutrophil, labelled with DiI, was photobleached and fluorescence recovery recorded in 100nm square subdomains, 500nm apart as shown in the images below. Recovery from the entire region (FRAP) and from the subdomains (sdFRAP) are shown, together with half times of recovery from equally spaced subdomains from the raw data. The graphs show the relationship for all subdomains taken from 2 successive photobleaches. The insert shows the untreated data and the main graph the relationship between time and the square of the distance with the fitted linear regression ( R^2^=0.97).

Thus the diffusion coefficient, D is proportional to the square of the distance travelled (x^2^) divided by the time taken (τ): D ∝ x^2^/(τ)

If x^2^ is the actual mean square displacement in time τ, and y^2^ is the apparent mean square displacement on a wrinkled surface in time, τw and the diffusion constant D for the molecule is unchanged

y^2^/ x^2^=τw/τ

y/x can be taken as a measure of the wrinkledness of the surface and is calculated from
 **√**(τw/τ)

Since the area of a smooth and wrinkled surface is proportional to the pathlength squared, τw/τ gives a measure of the wrinkledness of the surface between the photobleach front and the subdomain; and is used in this paper as the topographical index (Ti).

Thus Ti= τw/τs , where τw is the characteristic time for diffusion to the subdomain on the cell surface (wrinkled) and τs is the characteristic time for diffusion to the subdomain on a smooth surface.

**C. Membrane wrinkles after osmotic shrinking**

Before shrinking, the actual surface area (SA) of the cell is w.4πR^2^, where R is apparent radius of the cell and w is the factor by which wrinkled membrane increases that area.

After shrinking, the surface area (SA) = W.4πr^2^ where r is apparent new radius and W is the factor by which wrinkled membrane increases that area.

Since the actual SA does not change during shrinking:

w.4πRr^2^ = W.4πr^2^  or **W/w =** (**R/r)^2^**

**D. Estimating wrinkled morphology by SEM**

The percentage coverage by surface features (SF) visible as bright highlights in the SEM images was quantified. Pixels on the SEM images of cells which exceeded a threshold, set to distinguish high points on the cell surface, gave a measure of the percentage of surface features. Image J (NIH) was used with the threshold function. The coverage of high intensity features on individual neutrophils was calculated within a field which excluded cell edges. The same intensity threshold was employed for all cell images.

In populations of cells which were osmotically swollen, shrunk or to which deoxycholate was added, individual cells were quantified by both sdFRAP (to give a Ti value) and SEM (to give a percentage of surface feature coverage, SF). There was a correlation between the two estimates of cell surface topography as shown in the figure below.

**E. Atomic force microscopy of the surface topography of living neutrophils**

Atomic force microscopy (AFM) of living (non-fixed) neutrophils does not give an accurate representation of the micro-ridges and wrinkles on the cell body (see image below). This is in part because the body presents a “soft” and yielding surface for AFM probing and partly because over the time scale required for AFM image acquisition, the cell body topography is not static. Both effects result in a poorly defined topography on the cell body. Note that where the “skirt” around the cell has formed, this gives better resolution as the skirt is held in place by adhesion to the substrate and is less yielding to the AFM probe. We were thus not able to use AFM to investigate surface topography or to validate the sdFRAP technique using direct imaging by AFM.

*AFM image of living neutrophil attached to substrate by a “skirt” of thin cytoplasm. No surface structures can be imaged on the cell body, whereas the imaging of such detail can be seen on the skirt.*

**References**
1. Berg, H. C. *Random Walks in Biology*. (Princeton, 1977)
2. Bokshtein, B. S., Mendelev, M. I. Srolovitz, D. J. *Thermodynamics and Kinetics in Materials Science: A Short Course*. 167–171. (Oxford University Press, 2005).
3. Crank, J. *The Mathematics of Diffusion*. (Oxford University Press, 1980).
